# Drug-Induced Lipid Remodeling in *Leishmania* Parasites

**DOI:** 10.3390/microorganisms9040790

**Published:** 2021-04-09

**Authors:** Sneider Alexander Gutierrez Guarnizo, Elena B. Tikhonova, Masoud Zabet-Moghaddam, Kai Zhang, Carlos Muskus, Andrey L. Karamyshev, Zemfira N. Karamysheva

**Affiliations:** 1 Department of Cell Biology and Biochemistry, Texas Tech University Health Sciences Center, Lubbock, TX 79430, USA; sneider.gutierrez@udea.edu.co (S.A.G.G.); elena.tikhonova@ttuhsc.edu (E.B.T.); 2 Programa de Estudio y Control de Enfermedades Tropicales, Facultad de medicina, Universidad de Antioquia, Medellín 050010, Colombia; carlos.muskus@udea.edu.co; 3 Center for Biotechnology and Genomics, Texas Tech University, Lubbock, TX 79409, USA; masoud.zabet@ttu.edu; 4 Department of Biological Sciences, Texas Tech University, Lubbock, TX 79409, USA; kai.zhang@ttu.edu

**Keywords:** *Leishmania*, leishmaniasis treatment, antimony resistance, lipidomic, lipid remodeling, resistance phenotypes, therapeutic targets, drug resistance biomarkers

## Abstract

*Leishmania* parasites efficiently develop resistance against several types of drugs including antimonials, the primary antileishmanial drug historically implemented. The resistance to antimonials is considered to be a major risk factor for effective leishmaniasis treatment. To detect biomarkers/biopatterns for the differentiation of antimony-resistant *Leishmania* strains, we employed untargeted global mass spectrometry to identify intracellular lipids present in antimony sensitive and resistant parasites before and after antimony exposure. The lipidomic profiles effectively differentiated the sensitive and resistant phenotypes growing with and without antimony pressure. Resistant phenotypes were characterized by significant downregulation of phosphatidylcholines, sphingolipid decrease, and lysophosphatidylcholine increase, while sensitive phenotypes were characterized by the upregulation of triglycerides with long-chain fatty acids and a tendency toward the phosphatidylethanolamine decrease. Our findings suggest that the changes in lipid composition in antimony-resistant parasites contribute to the physiological response conducted to combat the oxidative stress unbalance caused by the drug. We have identified several lipids as potential biomarkers associated with the drug resistance.

## 1. Introduction

*Leishmania* species are the causal agent of leishmaniasis, a complex tropical disease considered to be the second most epidemiologically important after malaria, with more than 12 million infected people, 0.9 to 1.6 million new cases, and 20,000 to 30,000 deaths each year [1,2]. *Leishmania* adopts two different stages in the life cycle. The extracellular promastigote stage adapted to survive in the phlebotomine insect vector, and the intracellular amastigote stage adapted to survive inside of the macrophages. The geographical distribution, coinfection, host immune response, and *Leishmania* species/strains involved are associated with a wide range of different clinical outcomes including cutaneous, mucocutaneous and visceral forms [3].

Leishmaniasis’s clinical variability contributes to the difficulty of developing a unique efficient therapy. Historically, pentavalent antimonials (Sb^V^) have been used as the main leishmaniasis treatment worldwide for more than six decades [4]. It is commonly accepted that Sb^V^ works as a prodrug being reduced to trivalent antimony (Sb^III^). Sb^III^ induces oxidative stress [5,6]; inhibits the glycolytic pathway and fatty acid β-oxidation [7]; interferes with the purine salvage pathway [8]; inhibits the DNA topoisomerase I [9]; and competes with zinc (Zn^II^) for its binding to the CCHC and CCCH zinc finger domains [10]. However, antimony’s mode of action is not completely understood.

Unfortunately, the parasites have acquired resistance to Sb^V^ reaching treatment failure rates of over sixty percent [11]. Several mechanisms including drug activation, sequestration, uptake, and efflux have been found connected to antimony drug resistance [12,13]. Parasites can block the drug uptake by downregulation of aquaporin 1 (AQP1) [14]. They can also sequester drug via action of thiol–metal conjugates [15]. Alternatively, ATP-binding cassette (ABC) transporters might contribute to the increase in drug efflux [16]. Despite the progress made to understand the molecular mechanisms of antimony resistance our knowledge about the details of the mechanisms still remains limited and fragmentary.

Additionally, though several alternative drugs have been proposed including miltefosine, paromomycin, and liposomal amphotericin B, the parasites have shown the ability to generate resistance to all treatments [13,17,18,19,20]. In the absence of an effective vaccine, chemotherapy remains a major treatment option to control leishmaniasis. Nevertheless, with the rise of resistance events, drug toxicity, and the slow development of new therapies there is an urgent need to differentiate the antimony resistance phenotypes and optimize leishmaniasis treatment.

The understanding of molecular mechanisms of drug resistance and differentiation of antimony resistant phenotypes by biomarkers/biopatterns can contribute to the prediction of the clinical outcomes and avoid toxic drug treatment of patients infected with resistant strains, in which case the drug is inefficient. It will also help to the development of a personalized medical approach in leishmaniasis treatment. Several efforts have been conducted in this direction mainly at the genomic, transcriptomic, and proteomic levels. The data indicate that genetic variations and gene-dosage modifications as well as global changes at the mRNA and protein level occur during the antimony resistance development [21,22]. In contrast to analysis based on techniques such as next-generation sequencing (NGS), studies analyzing the metabolome and lipidome which are commonly recognized to be more representative of the cellular phenotype are less abundant in the field of antimony resistance, but they have been increasing thanks to more recent bioinformatics developments [23,24]. Consistently, these studies have shown that the redox metabolism, amino acid metabolism, and a lipid remodeling are commonly associated with antimony resistance phenotypes [12,25,26,27,28,29]. Nevertheless, the connection between the lipid adaptation and the antimony resistance mechanisms remains unknown.

At the global physiological level, lipids are quite diverse and representative molecules since they play important roles in several cellular processes. Lipids are involved in energy metabolism, energy storage, cell membranes composition, membrane trafficking, cellular signaling, cell proliferation, calcium homeostasis, and autophagy [30,31,32]. As a consequence, studying the lipidome, the complete set of lipids, offers the opportunity to analyze the physiological adaptation associated with drug-resistant phenotypes [33].

Lipidomic studies have been successfully completed for the detection of lipid biomarkers in other infectious diseases. Lipoteichoic acid has been used as predictor of the outcome for clinical sepsis [34]. The phosphatidylinositol-3-phosphate levels have been shown to be predictive of artemisinin resistance in both clinical and engineered in the laboratory Plasmodium parasites [35]. More recently, lipoarabinomannan has been suggested as a biomarker for tuberculosis diagnosis [36]. In leishmaniasis, components of the Kennedy pathway have been proposed as biopatterns for the differentiation of miltefosine resistant *Leishmania* parasites [37]. An altered sterol biosynthesis is one of the main amphotericin B resistance marks [38]. Furthermore, the fatty acids 18:1 Δ9c and 20:4 Δ5,8,11,14 have been preliminary suggested as potential biomarkers for nitric oxide and Sb^III^ resistance in *L. chagasi* and *L. amazonensis* [39]. However, the evidence for specific lipid changes during the development of antimony resistance is still very poor and limited; so, given the biological complexity of *Leishmania spp*., more studies are required to detect consistent and reliable lipid’s biomarkers/biopatterns of the antimony-resistant phenotypes.

The present study aims to identify potential lipid biomarkers/biopatterns related to Sb^III^-resistance through the differential analysis of the lipid composition between a Sb^III^-sensitive strain and a strain selected in vitro for Sb^III^ resistance. Our data demonstrate that the Sb^III^-resistance development involves a lipid remodeling. The lipidomic profiles effectively differentiate the Sb^III^-sensitive and Sb^III^-resistant phenotypes both in the absence and presence of the drug. The differences in lipid composition suggest that Sb^III^-resistant parasites trigger a specific lipid remodeling which can be connected to a more efficient oxidative stress response required to combat the drug.

## 2. Materials and Methods

### 2.1. Reagents

Chloroform (CAS: 67-66-3, Sigma-Aldrich, St. Louis, MO, USA), methanol (CAS: 67-56-1, Fisher Chemical, Hampton, NH, USA), formic acid (CAS: 64-18-6, Fisher Chemical), ammonium formate (CAS: 540-69-2 Fisher Scientific), isopropanol (67-63-0, Fisher Chemical), Schneider insect medium (S0146 Sigma Aldrich), and potassium antimony (III) tartrate trihydrate (CAS: 28300-74-5, Sigma Aldrich).

### 2.2. Parasites Culturing and Drug Treatment

The *Leishmania tropica* antimony sensitive wild type strain (WT) and isogenic antimony highly resistant strain (HR) generated on the base of the WT strain were used in this study. WT and HR strains have a half maximal effective concentration (EC50) of 10.4 ± 0.6 and 631.7 ± 73.7 µg/mL, respectively. The Sb^III^ or final drug product of Sb^V^ which acts as a prodrug, was administered as potassium antimony (III) tartrate trihydrate. Both sensitive and resistant strains were independently grown either in presence or absence of Sb^III^ generating four different experimental conditions, each one with three independent biological replicates.

In each case, the promastigotes were seeded at 5 × 10^5^ promastigotes/mL in a final volume of 50 mL of Schneider insect medium supplemented with 10% of Fetal Bovine Serum (FBS), 10 units/mL Penicillin, and 0.1 mg/mL of Streptomycin. After 36 h of growing, the Sb^III^ was added to the respective group at a final concentration of 100 µg/mL. The total time of drug exposure was 12 h, time in which most of sensitive parasites remain alive, allowing the isolation of intracellular lipids both in sensitive and resistant parasites. Once the drug exposure time was completed the parasites were harvested by centrifugation at 1800× *g*, 4 °C for 5 min and washed twice in 50 mL of cooled Dulbecco’s phosphate buffered saline (DPBS), using the same settings. The number of harvested parasites were normalized by the OD600 and a total of 4 × 10^8^ parasites were pelleted per condition. A second normalization by the total protein content was completed using the Bradford protein assay before the lipid extraction [40].

### 2.3. Lipid Extraction

The cell pellets were resuspended in 100 µL of water, followed by the addition of 400 µL of methanol plus 10 µL of EquiSPLASH™ LIPIDOMIX^®^ Quantitative Mass Spec Internal Standard according the protocol [41]. The mixture was homogenized using bead beater for 3 cycles of 30 s. The mixture was left on ice for 30 min, 400 µL of chloroform was then added and vortexed for 2 min. Next, 200 µL of water was added and samples were vortexed for another 2 min followed by centrifugation for 15 min to initiate phase separation. 300 µL of organic phase was collected for each sample and dried down. The dried samples were resuspended in methanol/chloroform (50:50) prior injecting into ultra-performance liquid chromatography coupled to tandem mass spectrometry system (UPLC-MS/MS).

### 2.4. UPLC-MS/MS

The UPLC-MS/MS was performed using Q-Exactive HF (Thermo Fisher Scientific) mass spectrometer with a heated electrospray ionization source (HESI) probe as described [42]. The HESI probe was operated at a spray voltage of 3.8 kV in the positive (+) mode and at 2.8 kV in the negative (−) mode with capillary temperatures of 300 °C (+) or 310 °C (−), respectively, sheath gas flow of 50 arbitrary unit, auxiliary gas flow of 10 arbitrary unit, and spare gas flow of 1 arbitrary unit.

The chromatographic separation of lipid samples was performed on Vanquish LC system using an Acquity UPLC BEH C18 Column, 130 Å, 1.7 µm, 2.1 mm × 100 mm, 1/pk from Waters. A 28 min gradient was used for separation using solvent a (water, 0.1% formic acid, 5 mM ammonium formate) and solvent b (methanol/isopropanol, 85/15, 0.1% formic acid, 2 mM ammonium formate). The gradient started with 35% solvent b, followed by increase to 60% b in 4 min and then increased to 85% b in 8 min and another increase to 100% b in 9 min. The gradient maintained at 100% b for another 3 min and finally decreased to 35% b in 0.1 min and stayed for another 4 min at 35% b solvent for the equilibration of the column. The flow rate was kept at 0.4 mL/min throughout the run and the column temperature was at 50 °C.

The mass spectrometry analysis was done in both positive and negative ion mode for each individual sample. Data-dependent acquisition mode with two scan events was employed for tandem mass spectrometry analysis (MS/MS). The first scan event was a full MS scan of 250–1200 *m*/*z* at a mass resolution of 120,000 full width at half maximum (FWHM) with an automatic gain control (AGC) target of 1 × 10^6^ and a maximum injection time (IT) of 100 ms. In the second scan event, 10 most intense ions detected in the first scan event were selected to perform Higher-energy collisional dissociation (HCD) MS/MS with resolution of 30,000. An elevated normalized collision energy (NCE) 25–30% was applied and the dynamic exclusion was set at 8 s.

### 2.5. Internal Normalization and Relative Lipid Quantitation

LipidSearch™ Software (4.1 Thermo Scientific, Waltham, MA, USA) was used for lipid identification. The acquired tandem spectra (MS and MS^2^) were searched against in silico predicted spectra of various lipid classes which includes more than 1.5 million lipid ions and their predicted fragment ions. The mass tolerance for the precursor (MS) and MS/MS product were kept at 3 ppm and 5 mDa, respectively. The MS/MS similarity score threshold was set at 5. The potential adductions included hydrogen, sodium, and ammonium for the positive ion mode (+) and hydrogen loss and formate for the negative ion (−) mode were predicted. Subsequently, the data in ESI+ and ESI– modes were merged and the dataset containing the area under peaks values equivalent to the lipid concentration were used to univariate and multivariate statistical analysis downstream.

The analysis was performed in three stages. (1) Peak detection, to discriminate reliable peak signals from noise. (2) Lipid identification was completed integrating the comprehensive ID and scoring algorithms. Comprehensive ID algorithm was used for product ion scans and discriminate each lipid by matching the predicted fragmentation pattern stored in the database, while Scoring algorithms were used to filter out lower probability results in identification. (3) Alignment and quantitation, Semi-quantification of lipids was performed by the normalization of lipid peak area to appropriate lipid internal standard (EquiSPLASH™) and also the number of cells. Prior to calculate the area under peak, lipid peaks alignment was done within a retention time window (0.1 min) across the sample sets for both ESI+ and ESI− ion modes and the results were merged into a single report. Identified lipids were then quantified by detecting their precursor ions from full-scan MS and integrating extracted ion chromatograms (XIC). Accurate peak areas were computed by denoising and smoothing the peak profiles prior to separating any partially overlapped peaks. The obtained area under peak values by XIC was then used for any further data analysis.

### 2.6. Data Normalization

The lipid peaks area values were processed in Metaboanalyst 4.0 software for data normalization. Data were filtered by the interquartile range (IQR), the samples were normalized using the probabilistic quotient normalization (PQN) and finally the data were log transformed and scaled by the Pareto method [43,44].

### 2.7. Lipid Class and Statistical Analysis

The distribution of the peak area values within different detected lipid classes was plotted using the function lipid class of the lipidr package of Bioconductor to explore the general lipidome coverage (https://github.com/ahmohamed/lipidr; accessed date March 23, 2021). For differential lipid expression analysis, the univariate analysis included the volcano plot for the detection of differently expressed lipid ions filtering by the *p*-value cutoff ≤ 0.05 and the absolute fold change cutoff ≥ 2. The multivariate analysis included both unsupervised and supervised methods. The unsupervised analysis for both outliers and clustering detection, was performed by a principal component analysis (PCA) [45]. The supervised analysis included the orthogonal partial least squares discriminant analysis (OPLS-DA) [46]. For each OPLS-DA model the R2Y (the percentage of variation explained by the model), Q2 (the predictive ability of the model) and one thousand permutation *p* values were calculated [47]. An S-plot which reveals the contribution of each variable to the predictive component was created for each OPLS-DA model. The S-plot is a correlation between the p1 or modelled covariance and p(corr)1 or modelled correlation, representing the lipid’s magnitude and reliability, respectively [48]. The model’s estimator bias were filtered by Jack-Knifing (JC) uncertainties method [49]. The four top variables with higher magnitude and reliability were prioritized as potential biomarkers.

## 3. Results

### 3.1. Design of the Lipidomic Experiment

**Figure 1:**
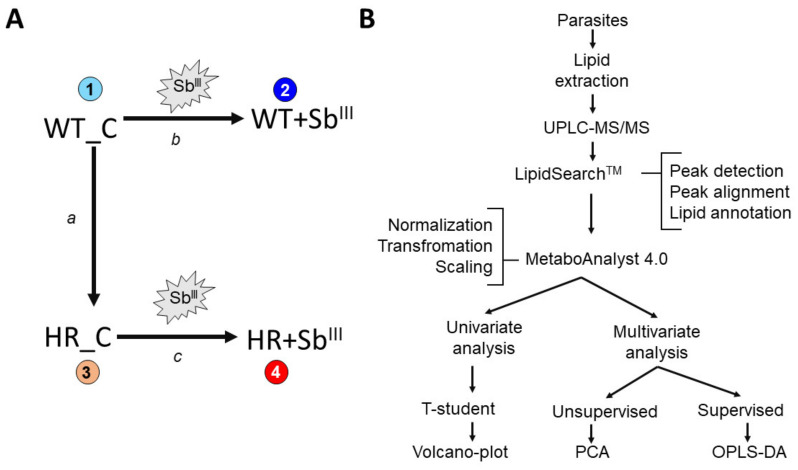
Schematic representation of the experimental design. (**A**) Four experimental conditions were evaluated (1–4), each one with three independent biological replicates. Experimental condition 1: Wild type strain control (WT_C), without Sb^III^ pressure. Experimental condition 2: wild type strain growing under Sb^III^ pressure (WT+Sb^III^). Experimental condition 3: antimony highly resistant strain control (HR_C), without Sb^III^ pressure. Experimental condition 4: antimony highly resistant strain growing under Sb^III^ pressure (HR+Sb^III^). Three dual comparisons for differential lipid expression were performed (a–c). (a) Basal changes in drug resistant strain in the absence of drug, by the comparison of resistant strain HR_C versus sensitive WT_C in the absence of Sb^III^ drug. (b) Sensitive strain responding to Sb^III^ by the comparison of WT+Sb^III^ versus WT_C. (c) Highly resistant strain responding to Sb^III^ by the comparison of HR+Sb^III^ versus HR_C. (**B**) Summarized workflow for lipidomic analysis from lipid extraction to statistical analysis including the integration of the LipidSearchTM (Thermo Scientific) and metaboanalysit 4.0 software. Volcano plots were used for the differential lipid expression analysis at the univariate method while principal component analysis (PCA) and Orthogonal partial least squares discriminant analysis (OPLS-DA) for multivariate approach.

It is expected that resistant parasites acquire phenotypic changes some of which can persist in the absence of the drug pressure while others can be only detected when the parasites are growing under antimony exposure. Under drug pressure both sensitive and resistant parasites actively induce a physiological response conducted to combat the antimony. However, this response is expected to be different between both phenotypes and can be a determinant of the parasite’s survival. Therefore, in order to examine differences and separate responses associated with drug resistance, both the antimony sensitive and resistant isogenic parasites were grown with and without antimony pressure (Figure 1A). Incubation with drug was performed for 12 h since it allows to keep both sensitive and resistant strains alive and it is sufficient to generate response. All experimental conditions were tested in three independent biological repeats. LipidSearchTM (Thermo Scientific) and MetaboAnalyst 4.0 software were used to analyze the lipidome data (Figure 1B). Downstream, the following data analysis was performed: (a) to detect classes of lipids present in *L. tropica*; (b) compare basal differences in lipid composition in WT and HR strains without drug treatment; (c) examine response to antimony drug in sensitive and resistant parasites; and (d) identify the main lipids contributing to differentiate the antimony-resistant phenotypes.

### 3.2. Leishmania Lipidome Profile by UPLC–MS/MS Identifies Twenty Different Classes of Lipids

**Figure 2:**
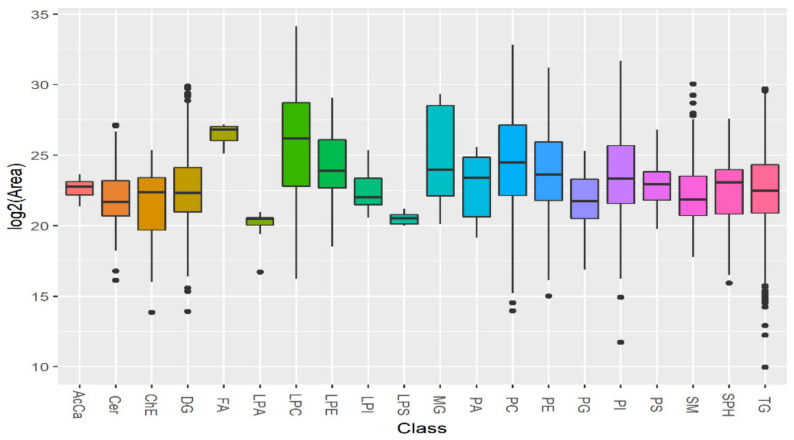
*Leishmania* lipidome profile identifies twenty different lipid classes. The signal intensity is represented as the integrated peak area transformed by binary logarithm function in the Y axis. The data from three biological repeats were analyzed. The lipidomic profile obtained by ultra-performance liquid chromatography coupled to tandem mass spectrometry (ULPC-MS/MS) allowed the detection of several lipid classes distributed in the X axis: acyl carnitine (AcCa), ceramide (Cer), cholesterol ester (ChE), diglyceride (DG), fatty acids (FA), lysophosphatidic acid (LPA), lysophosphatidylcholine (LPC), lysophosphatidylethanolamine (LPE), lysophosphatidylinositol (LPI), lysophosphatidylserine (LPS), monoglyceride (MG), phosphatidic acid (PA), phosphatidylcholine (PC), phosphatidylethanolamine (PE), phosphatidylglycerol (PG), phosphatidylinositol (PI), phosphatidylserine (PS), sphingomyelin (SM), sphingosine (SPH) and triglyceride (TG). The chart includes the lipid ions detected in both sensitive and resistant strains, merging lipids detected in positive and negative mode. In a typical boxplot, the vertical lines connect with the minimum and maximum values. The horizontal middle line of the boxes located between quartile 1 and quartile 3, represents the median, while the points at the ends represent the values considered outliers.

First, we determined what classes of lipids are present in sensitive and resistant *Leishmania tropica* strains. Our data analysis using UPLC–MS/MS detected 20 general classes belonging to three categories: sphingolipids, phospholipids and neutral lipids (Figure 2). Among them sphingolipids: sphingomyelin (SM), sphingosine (SPH), ceramide (Cer); phospholipids: lysophosphatidic acid (LPA), lysophosphatidylcholine (LPC), lysophosphatidylethanolamine (LPE), lysophosphatidylinositol (LPI), lysophosphatidylserine (LPS), phosphatidic acid (PA), phosphatidylcholine (PC), phosphatidylethanolamine (PE), phosphatidylglycerol (PG), phosphatidylinositol (PI), phosphatidylserine (PS); neutral lipids: acyl carnitine (AcCa), cholesterol ester (ChE), monoglyceride (MG), diglyceride (DG), fatty acids (FA), and triglyceride (TG) were detected. The lipid ion quantification represented as the integrated peak area showed that all identified lipid classes were detected in a high concentration, most of them with similar median concentration, while four lipid classes (AcCa, FA, LPA, and LPS) showed a particular low standard deviation represented as small size boxes.

### 3.3. The Antimony-Resistant Parasites Showed a Drastically Different Response in the Lipid Remodeling in Comparison with Sensitive Strain

**Figure 3:**
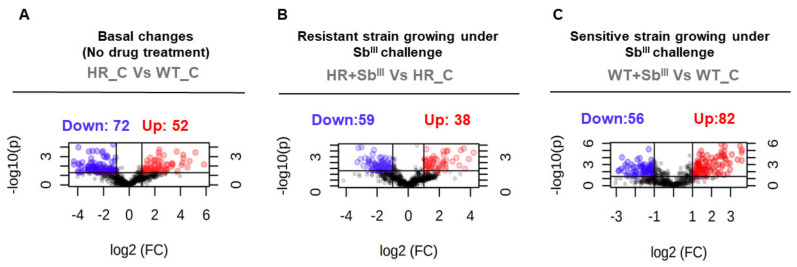
Volcano plots display significant differences at the lipid expression level in antimony resistant parasites. The volcano plots show the statistical significance (*p* value) transformed by the negative base 10 logarithm in the *Y*-axis versus the fold change (FC) transformed by the binary logarithmic function in the *X*-axis. The data from three biological repeats were analyzed. Statistically significant changes in lipids were detected in the antimony resistant strain under basal conditions in the absence of drug (**A**), in the antimony resistant strain responding to Sb^III^ (**B**), and in the antimony sensitive strain responding to Sb^III^ (**C**). The differentially expressed lipid ions were filtered by the *p*-value cutoff ≤ 0.05 and the absolute fold change cutoff ≥ 2. Significantly downregulated and upregulated lipid ions are highlighted with blue and red dots respectively while lipids with no significant changes are showed in black dots.

The differential lipid expression analysis was used to evaluate the changes in the lipid composition of sensitive and resistant parasites growing both without and under antimony pressure, evaluating four experimental conditions as shown on Figure 1. Three dual comparisons by univariate and multivariate statistical analysis were performed. First, we examined what are the basal differences in lipid composition in sensitive WT and resistant HR without drug exposition. Next, we tested the response to Sb^III^ in sensitive WT strain. Finally, we analyzed the response to antimony drug in resistant HR strain. The statistical results obtained by t-test are summarized in the Supplementary Files S1 and S2. All three comparisons revealed significant changes in lipid composition as shown on volcano plots (Figure 3; Supplementary File S1). First of all, our analysis has shown that lipid composition is different in the resistant strain even in the absence of the drug: 52 lipid ions are upregulated and 72 are downregulated in comparison with sensitive WT (Figure 3A). In addition, both drug resistant and sensitive parasites revealed a substantial lipid remodeling in response to the drug evident from volcano plots, however, the number of upregulated and downregulated lipid ions was different in resistant parasites (Figure 3B,C).

### 3.4. Most of the Lipids Differentially Expressed in Antimony Resistant or Sensitive Leishmania Parasites Belong to Phosphatidylcholines and Triglycerides Class

**Figure 4:**
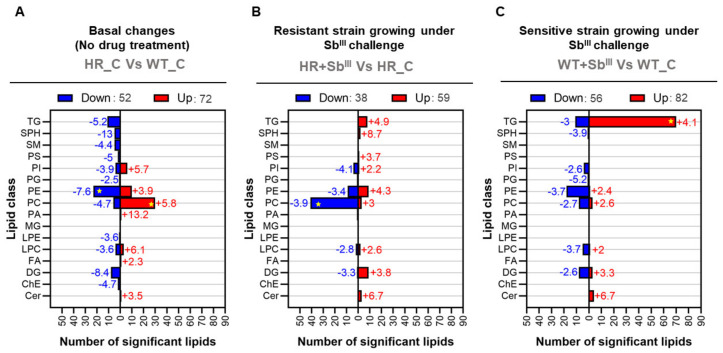
Phosphatidylcholines (PC) and triglycerides (TG) are the main lipid classes differentiating antimony resistant *Leishmania* phenotypes. All lipid classes detected are represented in the *Y*-axis and the number of lipids differentially expressed are represented in the *X*-axis. The data from three biological repeats were analyzed. The group of down regulated or up regulated lipids is highlighted in blue and red bars, respectively. The average fold changes are shown in front of each bar, and negative and positive values indicate if the lipids were detected to be decreases or increases, respectively. (**A**) Basal antimony resistant phenotype changes. (**B**) Resistant strain responding to Sb^III^. (**C**) Sensitive strain responding to Sb^III^. Duplicate signals coming from different adducts, multiple fragmentation or software lipid identification were filtered. Enriched lipid class with the highest number of differentially expressed lipids are highlighted with yellow star.

The differentially expressed lipids were grouped in all detected lipid classes except the monoglycerides. Notably, a few lipid classes: phosphatidylcholines, triglycerides, and even phosphatidylethanolamines, were highly enriched, encompassing most of the differentially expressed lipids (Figure 4). In differential lipid expression analysis, the fold changes represent the effect size, and the negative or positive fold changes values indicate downregulation or upregulation, respectively. The mean fold change (MFC) was calculated to estimate the magnitude of the changes per group of lipids. At the basal level when the drug is not present, the resistant parasites showed the downregulation of 23 PE (MFC: −7.6) and the upregulation of 30 PCs (+5.8) in comparison with sensitive parasites (Figure 4A). The resistant strain responding to the drug exhibited the downregulation of 41 PCs (MFC: −3.9) (Figure 4B). Contrary to resistant strain, the sensitive strain growing under antimony pressure showed a dramatically different response characterized by the upregulation of 70 triglycerides (MFC: +4.1) (Figure 4C; Supplementary File S2).

For this reason, a significant downregulation of phosphatidylcholines was consequently associated with *Leishmania’s* ability to resist the drug while a dramatic increase of triglycerides (TG) was mainly associated with the Sb^III^-sensitive phenotype. However, other lipid classes covering a smaller number of differentially expressed lipids were also affected and might have an important biological role.

Among the minor changes in terms of the number of significant lipids per lipid class, at the basal level 10 sphingolipids including 5 sphingomyelins (MFC: −4,4), and 5 sphingosines (MFC: −13), were downregulated in HR strain. A group of eight diglycerides showed an important mean decrease with an MFC of −8.4, but the group of sphingosines evidenced the greater decrease with an MFC of −13, while an individual phosphatidic acid exhibited a greater MFC of +13.2 (Figure 4A).

In the case of resistant strain growing under Sb^III^ exposure, 4 phosphatidylinositol (PI) and 41 phosphatidylcholines (PC) accounted for the lipid classes with the greater decrease with MFC of −4.1 and −3.9, respectively. On the contrary, a group of three ceramides (Cer), another type of sphingolipid showed a greater mean increase with the higher mean fold change (MFC: +6.7) (Figure 4B).

Finally, when the sensitive strain was grown under Sb^III^ pressure, the greater effect size in downregulated lipid classes was represented by a phosphatidylglycerol (PG) with a fold change of −5.2. Oppositely, four ceramides showed a greater mean increase with the higher MFC (+6.7), like the found in resistant parasites responding to antimonial (Figure 4C).

### 3.5. A Tendency toward the Downregulation of Phosphatidylcholine Is Associated with the Antimony Resistance Phenotype

**Figure 5:**
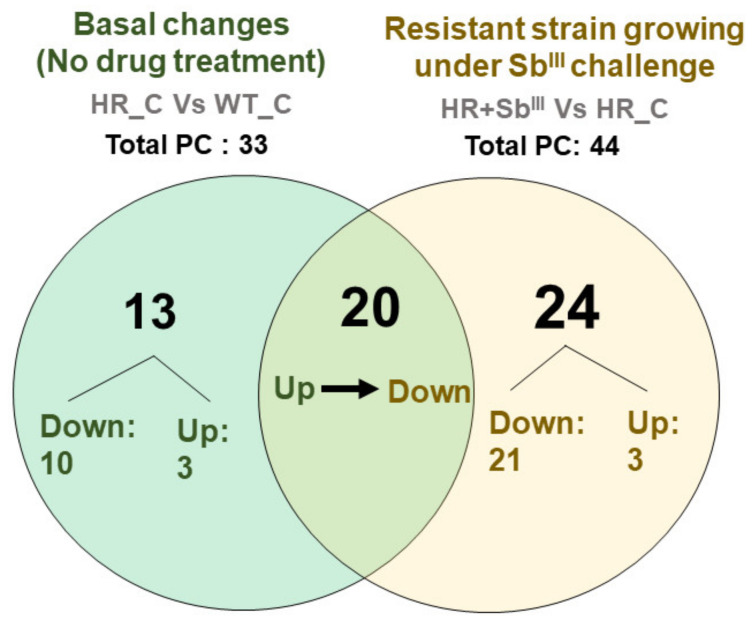
Phosphatidylcholine (PC) decrease is associated with the antimony resistant profile. The distribution of differentially expressed PCs is represented in a Venn diagram. Both the downregulated or upregulated PCs are plotted by two different comparisons: basal phenotypic changes in resistant strain in the absence of drug (green circle) and resistant strain responding to Sb^III^ (yellow circle). Total values correspond to total PC detected as significantly differentially expressed.

The comparative distribution of differentially expressed phosphatidylcholine detected in antimony resistant parasites before and after the drug challenge was summarized in a Venn diagram (Figure 5; Supplementary File S3). In the absence of the drug, a total of 33 phosphatidylcholine were differentially expressed in resistant parasites. Of which 13 PCs: PC(32:0), PC(32:1), PC(32:4), PC(34:1), PC(34:5), PC(36:1), PC(36:7), PC(38:0), PC(38:5), PC(38:7e), PC(42:10), PC(42:5), PC(44:10) were exclusives while 20 of them PC(30:1), PC(32:2), PC(33:2), PC(34:0), PC(34:3), PC(34:4), PC(35:4), PC(36:2), PC(36:4), PC(36:5), PC(36:6), PC(38:1), PC(38:2), PC(38:6), PC(40:3), PC(40:5), PC(40:7), PC(40:8), PC(40:9), and PC(42:1) were also detected as differentially expressed under drug pressure. Additionally, 20 PCs: PC(30:1), PC(32:2), PC(33:2), PC(34:0), PC(34:3), PC(34:4), PC(35:4), PC(36:2), PC(36:4), PC(36:5), PC(36:6), PC(38:1), PC(38:2), PC(38:6), PC(40:3), PC(40:5), PC(40:7), PC(40:8), PC(40:9), and PC(42:1) were only altered in response to the drug.

A total of 41 PCs were downregulated in the resistant strain after the drug treatment, it included all 20 PCs which were previously upregulated in the basal resistant phenotype (Figure 5; Supplementary File S3). The fact that about 87% (20/23) of the upregulated PCs became downregulated after drug treatment suggests that the PCs decline is required for an efficient drug response.

### 3.6. A Shift toward a Dramatic Upregulation of Triglycerides with More Long-Chain Fatty Acids Is Associated with the Antimony Sensitive Phenotype

**Figure 6:**
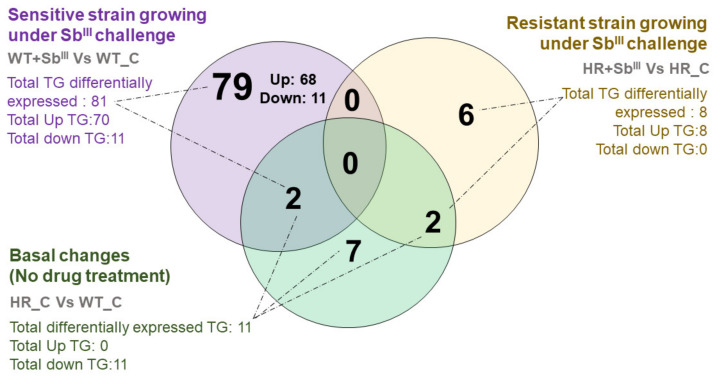
The profile of overexpressed triglycerides (TG) differs completely between antimony sensitive and resistant parasites. The distribution of differentially expressed TGs is represented in a Venn diagram. TGs distribution is plotted by three different comparisons: basal phenotypic changes in resistant strain in the absence of drug in comparison with sensitive (green circle), sensitive strain responding to Sb^III^ (purple circle) and resistant strain responding to Sb^III^ (yellow circle). The number of upregulated and downregulated triglycerides (TG) is represented per comparison.

Sensitive parasites had a completely different response. Triglycerides were the major class of lipids differentially expressed in sensitive strain as evident in Venn diagram (Figure 6) while resistant strain displayed most of the changes in phosphatidylcholines (Figure 5). A total of 81 triglycerides were differently expressed in the sensitive strain after Sb^III^ treatment of which only 11 were down-regulated while 70 triglycerides were strongly upregulated (TG(44:4), TG(46:5), TG(46:6), TG(46:7), TG(48:6), TG(49:5), TG(50:10), TG(50:4), TG(50:5), TG(50:6), TG(50:7), TG(50:8), TG(50:9), TG(52:6), TG(52:7), TG(52:8), TG(52:9), TG(54:10), TG(54:11), TG(54:12), TG(54:7), TG(54:8), TG(54:9), TG(55:8), TG(55:9), TG(56:10), TG(56:11), TG(56:12), TG(56:3e), TG(56:6), TG(56:7), TG(56:9), TG(57:10), TG(57:11), TG(58:11), TG(58:12), TG(58:13), TG(58:6e), TG(58:7e), TG(58:9), TG(59:10), TG(59:7e), TG(60:1), TG(60:10), TG(60:12), TG(60:13), TG(60:14), TG(60:2), TG(60:3), TG(60:4), TG(60:5), TG(60:7e), TG(60:9), TG(61:11), TG(61:4), TG(62:14), TG(62:15), TG(62:2), TG(62:4), TG(62:5), TG(62:7), TG(64:13), TG(64:16), TG(64:6), TG(64:7), TG(64:8e), TG(64:9), TG(66:7), TG(66:8), and TG(66:9)), of this 68 were exclusively detected in sensitive parasites treated with the drug, while 2 additional triglycerides were also detected as differently expressed in the basal changes. Oppositely, the resistant strain treated with Sb^III^, showed the upregulation of only eight different TGs, which were also different from the upregulated in sensitive parasites. Additionally, in the basal changes when the drug was not present, 11 triglycerides (TG) were differentially expressed, all of them detected as down-regulated (Figure 6, Supplementary File S4).

**Figure 7:**
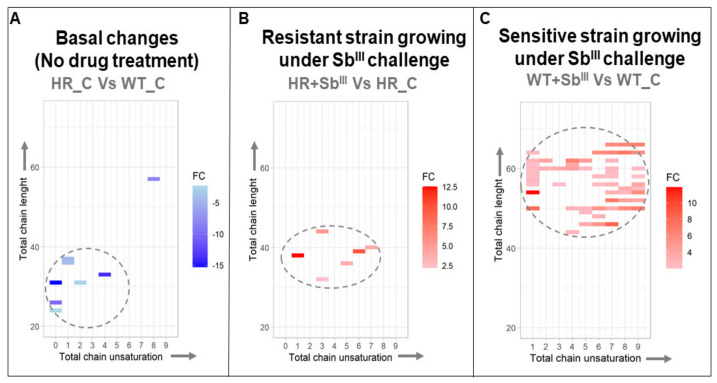
The upregulation of triglycerides with long-chain fatty acids is correlated with the antimony sensitive phenotype. The data from three biological repeats were analyzed. The charts represent the log 2-fold change of differentially expressed triglycerides (gradient color bar), distributed by the total chain lengths (*Y*-axis) and total unsaturation levels of fatty acids contained in triglycerides (*X*-axis). (**A**) Basal antimony resistant phenotype changes. (**B**) Resistant strain responding to Sb^III^. (**C**) Sensitive strain responding to Sb^III^. The downregulated and upregulated triglycerides (TGs) are represented by blue and red cells, respectively. Dotted lines highlight the main distribution of TGs per comparison.

In order to characterize in more details 68 upregulated triglycerides (TG) detected in sensitive parasites under drug challenge (Figure 6, purple circle) and establish if they were structurally different from 11 TGs specific to basal changes (Figure 6, green circle) or 8 TGs upregulated in resistant strain under drug pressure (Figure 6, yellow circle), we analyzed the total chain length and the total unsaturation level (Figure 7).

A clear structural differentiation was detected in triglycerides conformation across the experimental conditions. In the drug absence, the resistant strain exhibited the downregulation of short and mainly unsaturated triglycerides (Figure 7A); however, in response to the antimony, the resistant parasites changed their response toward the upregulation of more unsaturated triglycerides with slightly longer chains of fatty acids (Figure 7B). Particularly, the group of the upregulated triglycerides detected when the sensitive strain was treated with the drug, showed a shift towards a dramatic upregulation of triglycerides with very long-chain fatty acids between 44 and 66 carbons, which was not detected in the other comparisons (Figure 7C; Supplementary File S5).

### 3.7. The Changes in Lipid Composition Allow the Differentiation of Antimony Resistant Phenotypes by Multivariate Analysis

**Figure 8:**
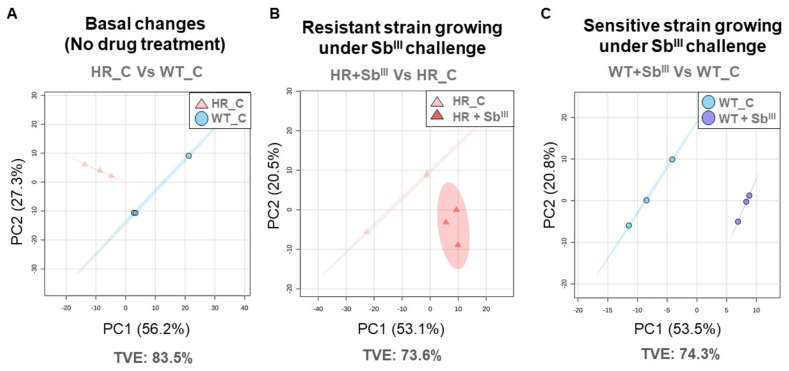
Unsupervised multivariate analysis showing a clear sample grouping per class. The PCA score plots are represented by individual comparison. The data from three biological repeats were analyzed. (**A**) PCA score plot of the basal antimony resistant phenotype changes explaining 83.5% of the total variance. (**B**) PCA score plot of the resistant strain responding to Sb^III^, explaining 73.6% of the total variance. (**C**) PCA score plot of the sensitive strain responding to Sb^III^, explaining 74.3% of the total variance. Each individual marker represents a biological replicate. Total variance explained (TVE) in summarized. The 95% confidence ellipse using Hotelling T-squared is represented.

Our results demonstrate dramatic changes in the lipid composition in drug resistant strain under the basal conditions and in response to the drug (Figure 4, Figure 5, Figure 6 and Figure 7). Next, we intended to explore what lipids have a potential to serve as biomarkers to distinguish drug resistant strain from the sensitive. The unsupervised analysis by PCA showed that the biological replicates were grouped per experimental condition without any outlier detected suggesting that the lipidomic profiles can be efficiently used to differentiate the resistant and sensitive phenotypes (Figure 8).

**Figure 9:**
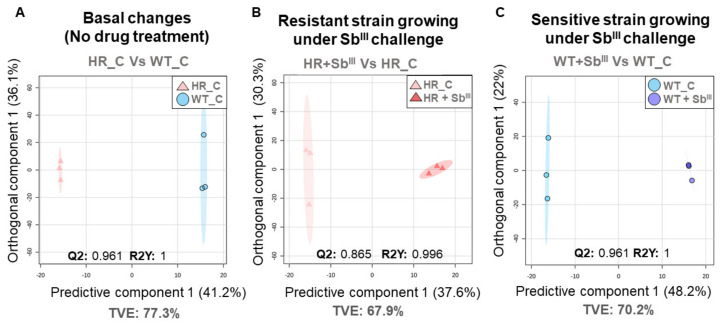
Supervised multivariate analysis indicating a clear inter-class discrimination. The score plot from Orthogonal partial least squares discriminant analysis (OPLS-DA) are represented by individual comparison. The data from three biological repeats were analyzed. (**A**) OPLS-DA score plot for the basal antimony resistant phenotype changes. (**B**) OPLS-DA score plot for the resistant strain responding to Sb^III^. (**C**) OPLS-DA score plot for the sensitive strain responding to Sb^III^. All models were successfully validated by one thousand permutations getting optimal Q2 and R2Y scores without any evidence of overfitting. Each individual marker represents a biological replicate. Total variance explained (TVE) in summarized. The 95% confidence ellipse using Hotelling T-squared is represented.

The supervised analysis by OPLS-DA model were carried out per each stablished comparison, in each case both the goodness of fit (R^2^Y) and goodness of prediction (Q^2^) had optimal and significant values (R^2^Y and Q^2^ ≥ 0.6, R^2^Y ≠ Q^2^ < 0.3) confirming that the resistant parasites can be differentiated in their lipid composition from sensitive parasites under basal conditions and during the drug treatment (Figure 9).

**Figure 10:**
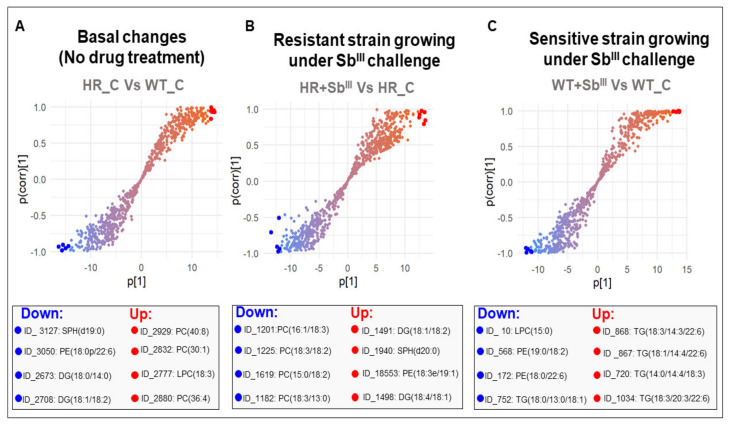
S-plots from the OPLS-DA models, for the detection of potential biomarkers. The S-plot is a scatter plot of the p[1] (magnitude effect) vs p(corr)[1] (reliability) vectors of the predictive component. (**A**) S-plot for the basal antimony resistant phenotype changes. (**B**) S-plot for the resistant strain responding to Sb^III^. (**C**) S-plot for the sensitive strain responding to Sb^III^. The lipids with higher reliability and magnitude effect across the group discrimination are highlighted at the S-plot tails. The top four of both downregulated and upregulated lipid ions are highlighted with blue and red dots respectively.

To identify potential biomarkers in terms of the lipids that mainly contributed to group separation, corresponding S-plots were constructed from OPLS-DA where the coordinates in the lower-left quadrant correspond to the lipids significantly decreased while those in the upper-right quadrant represent the lipids significantly increased (Figure 10; Supplementary File S6). The lipids with higher magnitude effect and reliability were suggested as potential biomarkers. It included 4 diglycerides, 2 sphingosines, 4 phosphatidylethanolamines, 7 phosphatidylcholines, 2 lysophosphatidylcholines, and 5 triglycerides (Figure 10). All them were also verified using univariate statistical analysis (Supplementary File S7).

More details about identified potential biomarkers are shown in Table 1. Most dramatic changes included downregulation of SPH(d19:0) and PE(18:0p/22:6) in the resistant parasites under the basal conditions and upregulation of DG(18:1/18:2) in response to drug. The downregulation of two PEs and upregulation of several TGs were the hallmarks of drug susceptibility in sensitive strain (Table 1).

## 4. Discussion

### 4.1. Phosphatidylcholine (PC) Decrease as a Possible Mechanism to Efficiently Combat Oxidative Stress Caused by Antimony Drug in Leishmania Resistant Parasites

Phosphatidylcholine (PC) is a major cell membrane constituent and precursor of important second messengers. In *Leishmania* parasites, PC synthesis can occur via the CDP-choline pathway (Kennedy pathway), the N-methylation of phosphatidylethanolamine (PE), and by the remodeling of exogenous phospholipids [50]. Particularly, while choline is a potential source of methyl donors, PC biosynthesis via PE methylation requires S-adenosylmethionine (SAM), a universal methyl donor involved in several roles in protozoan parasites [51]. SAM can participate in several interconnected pathways such as the methionine cycle, the synthesis of polyamines (spermidine, spermine), and the trans-sulfuration branch of homocysteine metabolism forming trypanothione (two molecules of glutathione joined by spermidine) [52]. The activation of the trypanothione metabolism has been widely documented as a mechanism of antimony resistance contributing to combat the oxidative stress caused by the drug. Thiols can produce conjugates thiol-Sb^III^ inhibiting the drug action, a process followed by the thiol-Sb^III^ efflux via exocytosis [53].

In this study, we hypothesize that antimony-resistant parasites under drug pressure can reprogram the phospholipid metabolism to partially reduce the phosphatidylcholine (PC) biosynthesis which could potentially increase the availability of methyl group donors to improve the thiol metabolism and optimize the oxidative/osmotic stress response. Interestingly, at the basal level, the PC increase was accompanied by a reduction in PE levels suggesting that in the absence of the drug the PC biosynthesis is increased via N-methylation of phosphatidylethanolamine (PE) (Figure 4A and Figure 5). The fact that most of the upregulated phosphatidylcholines (PC) in antimony resistant parasites were downregulated once antimony was applied (Figure 4B) strongly suggests that the PC storage could work as an adaptative strategy of some antimony-resistant parasites to release methyl group donors such as betaine via PC hydrolysis (degradation) once the drug is applied to combat oxidative stress caused by the drug.

**Figure 11:**
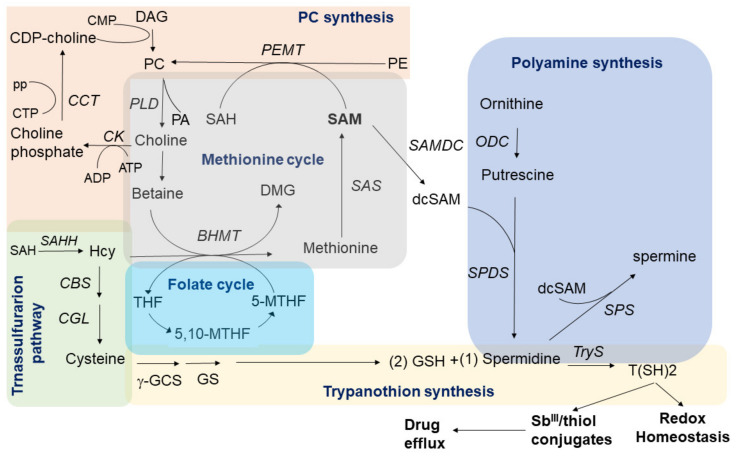
Summarized interconnection between PC synthesis, trans-sulfuration pathway, folate cycle, methionine cycle, polyamine pathway, and trypanothion synthesis. In antimony resistant parasites a decrease in PC biosynthesis and/or the activation of the PC hydrolysis via phospholipase D (PLD) can potentially contribute to increase the SAM levels and other methyl group donors, which can be addressed to feed the polyamine pathway and improve the trypanothione T(SH)2 synthesis. In *Leishmania*, T(SH)2 is the main antioxidant molecule and can interact with Sb^III^. It is commonly accepted that this complex can be transported via ABC (ATP-binding cassette) transporters and released during drug efflux. 5,10-MTHF: 5,10-Methylenetetrahydrofolate; 5-MTHF: 5-methyltetrahydrofolate; 5-MTHF: 5-methyltetrahydrofolate; ADP: Adenosine diphosphate; ATP: Adenosine triphosphate; BHMT: Betaine Homocysteine Methyltransferase; CBS: Cystathionine b-synthase; CCT: phosphocholine cytidylyltransferase; CEPT: choline/ethanolamine phosphotransferase; CGL: Cystathionine gamma-lyase; CK: Choline kinase; CMP: Cytidine monophosphate; CPT: CDP-choline phosphotransferase; CTP: Cytidine triphosphate; DAG: Diacylglycerol; dcSAM: decarboxylated S-adenosyl-L-methionine; DMG: Dimethylglycine; ODC: Ornithine decarboxylase; PA: phosphatidic acid; PC: phosphatidylcholine; PE: phosphatidylethanolamine; PEMT: Phosphatidylethanolamine methyltransferase; pp: pyrophosphate; SAH: S-adenosyl homocysteine; SAHH: S -adenosyl- L -homocysteine hydrolase; SAM: S-adenosyl-L-methionine; SAMDC: SAM decarboxylase; SAS: S-adenosylmethionine synthetase; SPDS: Spermidine synthase; SPS: Spermine synthase; T(SH)2: Trypanothione; THF: tetrahydrofolate; TryS: Trypanothione synthase.

Consequently, the availability of methyl donor groups can be also improved by their recovery via phosphatidylcholine (PC) hydrolysis/degradation. Although it is not clear if *Leishmania* parasites can recover methyl group donors from PC by hydrolysis, this alternative should also be considered. Phospholipase D (PLD) predominantly catalyzes the hydrolysis of PC (Figure 11), producing soluble choline and the signal molecule phosphatidic acid. Although phospholipase D gene coding has not been annotated in *Leishmania* genome, PLD activity has been detected experimentally in *Leishmania* parasites responding to osmotic stress, a common effect of antimonial in part due to oxidative stress [54].

The choline produced by PC hydrolysis can be potentially oxidized downstream to betaine or trimethylglycine, a methyl group donor known as a potent osmotic regulator [55]. The oxidation of choline to betaine links PLD activity to 1C metabolism through de novo synthesis of methionine from homocysteine. Methionine can be used to produce SAM via S-adenosylmethionine synthetase (SAS), an enzyme which have been consistently detected increased in antimony resistant parasites, contributing to the proposed hypothesis in which the antimony resistant parasites require to optimize the methyl group donors for an improved oxidative/osmotic stress response to combat the drug [52,56] (Figure 11).

Remarkably, other studies support this idea. Significant downregulation of 10 PCs were found in promastigotes of *L. donovani* clinical isolates with antimony-resistance [37]. In accordance, the up-regulation of the methyl donor 5-Methyltetrahydrofolate, a key component of the interconnected network of Methionine–Homocysteine–Folate metabolism, and the down-regulation of CDP choline, a PC precursor via CDP-choline pathway, were associated with the antimony resistance, supporting the idea that a PC decline and methyl donors increase are associated with the antimony resistance profile [29]. Additionally, the fact that under drug exposure, the antimony resistant parasites increased the diglycerides levels, support the idea that PCs biosynthesis via CDP-choline is declined in Sb^III^-resistant parasites, reducing the diglycerides intake to this pathway which can be detected as a diglycerides increase (Figure 4B and Figure 11).

### 4.2. The Upregulation of Triglycerides with More Long Chains of Fatty Acids (TG) in Antimony-Sensitive Parasites Is a Consequence of the β-Oxidation Inhibition Caused by the Drug

One of the first alterations reported in *Leishmania* parasites exposed to antimony, was the inhibition of fatty acid β-oxidation, the degradation process of fatty acids, the main components of triglycerides [57]. However, the antimony targets involved in β-oxidation inhibition are still unknown. Here, we found that the upregulation of triglycerides was correlated with the antimony-sensitive phenotype (Figure 4C). We hypothesize that the increase TGs is due to fatty acid β-oxidation inhibition caused by the drug in sensitive parasites.

This TGs upregulation involved triglycerides with particularly long chains of fatty acids (Figure 7C). In *Leishmania* parasites, the long-chain substrates are efficiently used as an energy source [7]. Other studies based on gas chromatography and mass spectrometry (GC-MS), have also found that the antimony exposure of *L. donovani* leads to an increase in long fatty acids [27].

Otherwise, there are evidences to suggest that a non-inhibition of the fatty acid β-oxidation is associated with an antimony-resistant profile. Not only genetic variations but also a predominant upregulation in proteins involved in fatty acid β-oxidation have been described in antimony resistant parasites [22,58,59]. Particularly, the long-chain fatty acyl-CoA ligase (LCFA) a key enzyme involved in β-oxidation of long fatty acids has been found to be differentially increased in antimony-resistant *Leishmania donovani* amastigotes by microarray analysis [60,61]. Furthermore, LCFA was detected to be upregulated at the protein level in antimony-resistant promastigotes of *L. infantum* [62].

Inevitably, the fatty acid β-oxidation as an oxidative process would contribute to increase in the free radical levels. It is probable that the antimony-resistant parasites, which commonly have an optimized oxidative stress response [12], can keep the β-oxidation activated without any oxidative stress unbalance or triglycerides (TG) accumulation, generating energy and carbon skeletons required to feed other pathways necessary to combat the drug and/or compensate the biological fitness.

**Figure 12:**
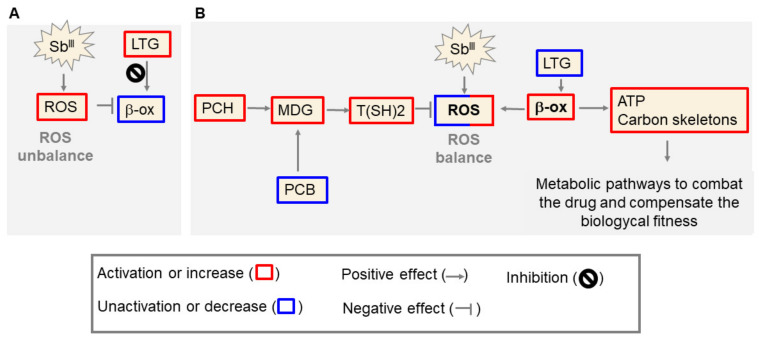
Model of antimony resistance in *Leishmania* parasites at the lipidomic level. (**A**) The antimony (Sb^III^) induces reactive oxygen species (ROS) production and β-oxidation (β-ox) inhibition, causing accumulation of triglycerides with more long chains fatty acids (LTG) and a poor energy metabolism. (**B**) To combat those alterations, resistant parasites rescue methyl donor groups (MDG) such as SAM and DMG. This strategy involves PC biosynthesis decrease (PCB) and/or the increase of PC hydrolysis (PCH). Higher MDGs levels improve the thiol (R-SH) metabolism and the downstream reaction of trypanothione T(SH)2 synthesis required to reach a ROS balance. The successful antioxidant response offers the opportunity for the controlled activation of pathways ROS generators, such as the β-ox. The β-ox can use the fatty acids from LTG to produce ATP and carbon skeleton, which are required to activate the metabolic pathways involved in both the drug control (drug sequestration, drug metabolism or drug efflux) and the biological fitness compensation.

Here we propose our model that in antimony resistant parasites the decline of PC is connected to an optimized stress response which could help to avoid the β-oxidation inhibition induced by the drug (Figure 12). Nevertheless, the components involved in the coordinating of the lipid metabolism reprogramming in response to the antimony remains to be clarified and more studies are required in this direction; especially because the biological functions of about 60% of protein-coding genes in *Leishmania* are still unknown.

### 4.3. Potential Biomarkers for the Differentiation of Antimony Resistant Leishmania Phenotypes

Our data analysis revealed that the PCs downregulation (PC(16:1/18:3), PC(18:3/18:2), PC(15:0/18:2), PC(18:3/13:0)) (Figure 10B) and the TGs upregulation (TG(18:3/14:3/22:6), TG(18:1/14:4/22:6), TG(14:0/14:4/18:3), and TG(18:3/20:3/22:6)) were consistent with the major response to the antimony by resistant and sensitive parasites, respectively. Therefore, these two groups of the lipids have been identified in our study as potential lipid biomarkers to differentiate the resistant and sensitive parasites. However, other lipid classes were also represented showing more complex patterns (SPH, LPC, and PE). In trypanosomatids, sphingosine is a type of sphingolipid involved in several roles including, cell signaling, calcium transport, and phosphatidylethanolamine (PE) biosynthesis [63,64]. Only two sphingolipids were selected as biomarkers (SPH(d19:0), SPH(d20:0)) making it difficult to associate it with any phenotype. However, after the differential lipid expression analysis, not only five sphingosines (SPH(d16:0), SPH(d17:0), SPH(d18:0), SPH(d19:0), and SPH(d20:0)) but also sphingomyelin (SM(d32:1), SM(d36:1), SM(d36:2D9), SM(d40:1), and SM(d42:2)), were downregulated in high resistant parasites at the basal level (Figure 4A).

Consistently, another study has shown that during the stationary growth phase and without antimony pressure sphingolipids are also less abundant in drug-resistant clones [29]. The authors claimed that the sphingolipids are less abundant in drug-resistant parasites since these molecules are consumed at a higher rate to fuel PE biosynthesis which is more abundant in the resistant parasites [29]. However, in our study, a dramatical PE upregulation was not detected in antimony resistant parasites.

Alternatively, sphingolipids can modulate the calcium fluxes through different mechanisms [65]. Calcium fluxes are also tightly correlated with oxidative stress and programmed cell death [66]. Even though, there is not enough evidence, is also probable that the remodeling of the sphingolipids profiles during antimony resistant development in *Leishmania* can contribute to the oxidative stress balance required to resist to the drug.

Phosphatidylethanolamine (PE) together with phosphatidylcholine (PC) are the most abundant phospholipids in eukaryotic cell membranes. Here, we detected that in response to the drug the PE levels in sensitive parasites trends clearly towards the downregulation (Figure 4C), while antimony resistant parasites showed a more balanced pattern between upregulated/downregulated PE (Figure 4B). However, as it was mentioned before, other study has showed that PEs are dramatically increased in drug-resistant clones from clinical isolates [29].

Of four PEs which were proposed as potential biomarkers, PE(18:3e/19:1) was increased in response to the drug in resistant parasites (Figure 10B), while PE(19:0/18:2) and PE(18:0/22:6) were decreased in sensitive parasites growing under antimony pressure (Figure 10C), supporting the idea that a tendency toward PE increase could have a role during the response to antimony in resistant parasites (Figure 4B,C).

The lysophosphocholines (LPC) are derived from partial hydrolysis of PC. LPC(18:3) was selected as a biomarker increased in resistant parasites at the basal level (Figure 10A), while LPC(15:0) was selected as a biomarker based on decrease in the sensitive strain treated with Sb^III^ (Figure 10C). The tendency toward more abundant LPCs in antimony-resistant parasites could support the previously discussed hypothesis that PC’s hydrolysis can take place as an alternative to improve the methyl group donor’s availability.

We also looked for some lipidic compounds exclusively or almost exclusively detected in sensitive or resistant phenotype independently if the drug is applied or not, which could not be detected by the orthogonal analysis. Probably, these molecules could be used even as more reliable biomarkers since they are independent of the Sb^III^ exposure. The putative 1,20-Eicosanediol and DG(34:0) were more abundant in resistant parasites, while DG(15:0/0:0/16:1n7), PE(40:6), DG(24:1n9/0:0/18:2n6) were more abundant in sensitive parasites (Supplementary File S8).

Together, these findings can be used as the base of future targeted studies addressed for the validation of the biomarkers-biopatterns associated with antimony resistance at the population level. Here we focused on an in vitro approach so more studies with clinical isolates will be required. These potential lipid biomarkers or their associated enzymes could potentially be used as predictors of the drug antimony efficiency during Leishmaniasis outbreaks. However, if the results obtained in this study is an exclusive phenomenon of *Leishmania tropica* or that could be extrapolated to other *Leishmania* species, must be investigated.

## 5. Conclusions

Our study demonstrates that the development of antimony resistance includes extensive and dynamic lipid remodeling. We proposed that particular lipids can be used for the differentiation of antimony resistant and sensitive phenotypes. We observed the differences in lipidomic profiles at both in the absence of drug and upon drug exposure. In the absence of the drug the main alterations in resistant parasites included the sphingolipids decrease and the PC increase possibly via PE methylation, as potential methyl group donor source to counteract oxidative stress. Under the drug treatment the long TG’s accumulation as a possible result of the β-oxidation and PC’s decay as an inducible response enhancing antioxidant response clearly separated the sensitive and resistant parasites, respectively.

Currently, it is not clear how some other differentially expressed lipids including sphingolipids, phosphatidylinositols (PI), phosphatidylserines (PS), phosphatidylglycerols (PG), etc., are associated with antimony resistance. Future studies are required in this direction. It would be also beneficial to explore if any of the lipids identified in connection to antimony drug resistance in the present study can serve as novel drug targets and lead to the development of new drugs to treat leishmaniasis. Finally, the employed strategy is a powerful approach to characterize *Leishmania’s* phenotypes and could be applied to identify the physiological adaptation of parasites responding to different stressors including other anti-*Leishmania* therapies.

## Figures and Tables

**Table 1:** List of potential lipid biomarkers for the differentiation of antimony resistant phenotypes in *Leishmania*.

ID	Lipid Ion	Polarity	RT	CalcMz	FC	*p*-Value	Category	Main Class	Comparison
3127	SPH(d19:0)	Positive	9.606341	316.321	−74.921	3.97 × 10^−3^	SL	PSL	Basal changes in resistant strain
3050	PE(18:0p/22:6)	Positive	17.03432	776.5589	−38.519	8.37 × 10^−4^	GP	GPE	Basal changes in resistant strain
2673	DG(18:0/14:0)	Positive	17.03642	586.5405	−32.643	1.36 × 10^−3^	GL	DRG	Basal changes in resistant strain
2708	DG(18:1/18:2)	Positive	16.97843	619.5296	−20.236	2.46 × 10^−3^	GL	DRG	Basal changes in resistant strain
2929	PC(40:8)	Positive	15.28111	830.5694	16.74	6.18 × 10^−3^	GP	GPC	Basal changes in resistant strain
2832	PC(30:1)	Positive	14.87514	726.5044	16.644	2.16 × 10^−3^	GP	GPC	Basal changes in resistant strain
2777	LPC(18:3)	Positive	10.05254	518.3241	13.912	1.99 × 10^−4^	GP	GPC	Basal changes in resistant strain
2880	PC(36:4)	Positive	15.87022	782.5694	7.685	9.10 × 10^−5^	GP	GPC	Basal changes in resistant strain
1201	PC(16:1/18:3)	Positive	14.89068	798.5291	−8.056	4.68 × 10^−4^	GP	GPC	Resistant strain growing under Sb^III^
1225	PC(18:3/18:2)	Negative	15.12758	764.5236	−8.372	4.98 × 10^−3^	GP	GPC	Resistant strain growing under Sb^III^
1619	PC(15:0/18:2)	Positive	15.43099	744.5538	−5.797	5.07 × 10^−3^	GP	GPC	Resistant strain growing under Sb^III^
1182	PC(18:3/13:0)	Positive	14.22137	758.4978	−4.878	1.86 × 10^−3^	GP	GPC	Resistant strain growing under Sb^III^
1491	DG(18:1/18:2)	Positive	16.97117	641.5115	18.504	1.37 × 10^−3^	GL	DRG	Resistant strain growing under Sb^III^
1940	SPH(d20:0)	Positive	10.43559	330.3367	11.92	6.18 × 10^−4^	SL	PSL	Resistant strain growing under Sb^III^
1853	PE(18:3e/19:1)	Positive	16.97688	762.5408	10.462	8.59 × 10^−4^	GP	GPE	Resistant strain growing under Sb^III^
1498	DG(18:4/18:1)	Positive	16.20263	637.4802	5.548	1.08 × 10^−3^	GL	DRG	Resistant strain growing under Sb^III^
10	LPC (15:0)	Positive	8.611845	526.315	−6.48	9.36 × 10^−5^	GP	GPC	Sensitive strain growing under Sb^III^
568	PE(19:0/18:2)	Positive	16.90759	758.5694	−7.406	8.40 × 10^−3^	GP	GPE	Sensitive strain growing under Sb^III^
172	PE(18:0/22:6)	Negative	16.58543	790.5392	−5.265	4.00 × 10^−4^	GP	GPE	Sensitive strain growing under Sb^III^
752	TG(18:0/13:0/18:1)	Positive	20.97317	841.7256	−5.992	8.90 × 10^−3^	GL	TRG	Sensitive strain growing under Sb^III^
868	TG(18:3/14:3/22:6)	Positive	19.10632	884.6763	11.242	2.26 × 10^−6^	GL	TRG	Sensitive strain growing under Sb^III^
867	TG(18:1/14:4/22:6)	Positive	19.3979	891.6473	12.049	8.53 × 10^−6^	GL	TRG	Sensitive strain growing under Sb^III^
720	TG(14:0/14:4/18:3)	Positive	18.52557	782.6293	8.3318	3.77 × 10^−5^	GL	TRG	Sensitive strain growing under Sb^III^
1034	TG(18:3/20:3/22:6)	Positive	20.30457	968.7702	10.236	2.01 × 10^−4^	GL	TRG	Sensitive strain growing under Sb^III^

ID, random identification number assigned per lipid ion; RT, retention time; CalcMz, calculated mass; FC, fold change; Category, Lipid Maps category; Main class, Lipid Maps main class; GP, Glycerophospholipids; GL, Glycerolipids; SL, Sphingolipids; GPC, Glycerophosphocholines; GPE, Glycerophosphoethanolamines; TRG, Triradylglycerols; DRG, Diradylglycerols; PSL, Phosphosphingolipids.

## Data Availability

The data presented in this study are available on request from the corresponding authors.

## Supplementary Materials

The following are available online at https://www.mdpi.com/article/10.3390/microorganisms9040790/s1. Supplementary File S1. Raw and normalized chromatography peak area intensities per lipid and comparison. Supplementary File S2. Downregulated and upregulated lipids per lipid class and comparison. Supplementary File S3. Venn diagram raw values of phosphatidylcholine distribution. Supplementary File S4. Venn diagram raw values of triglycerides distribution. Supplementary File S5. Raw data of triglycerides total length chain and unsaturation per experimental condition. Supplementary File S6. Raw OPLS-DA components per comparison. Supplementary File S7. Univariate analysis for the selected potential biomarkers. Supplementary File 8. List of putative lipids consistently discriminating antimony resistant parasites both under and without antimony challenge.
