# Supplementary material for: Drug-Induced Lipid Remodeling in Leishmania Parasites
Source: Microorganisms. 2021 Apr 9;9(4):790. doi: 10.3390/microorganisms9040790 (PMC8068835; doi:10.3390/microorganisms9040790)
Supplement: Supplementary file 1 [file microorganisms-09-00790-s001.zip › Supplementary files/Supplementary_fileS7.pdf]

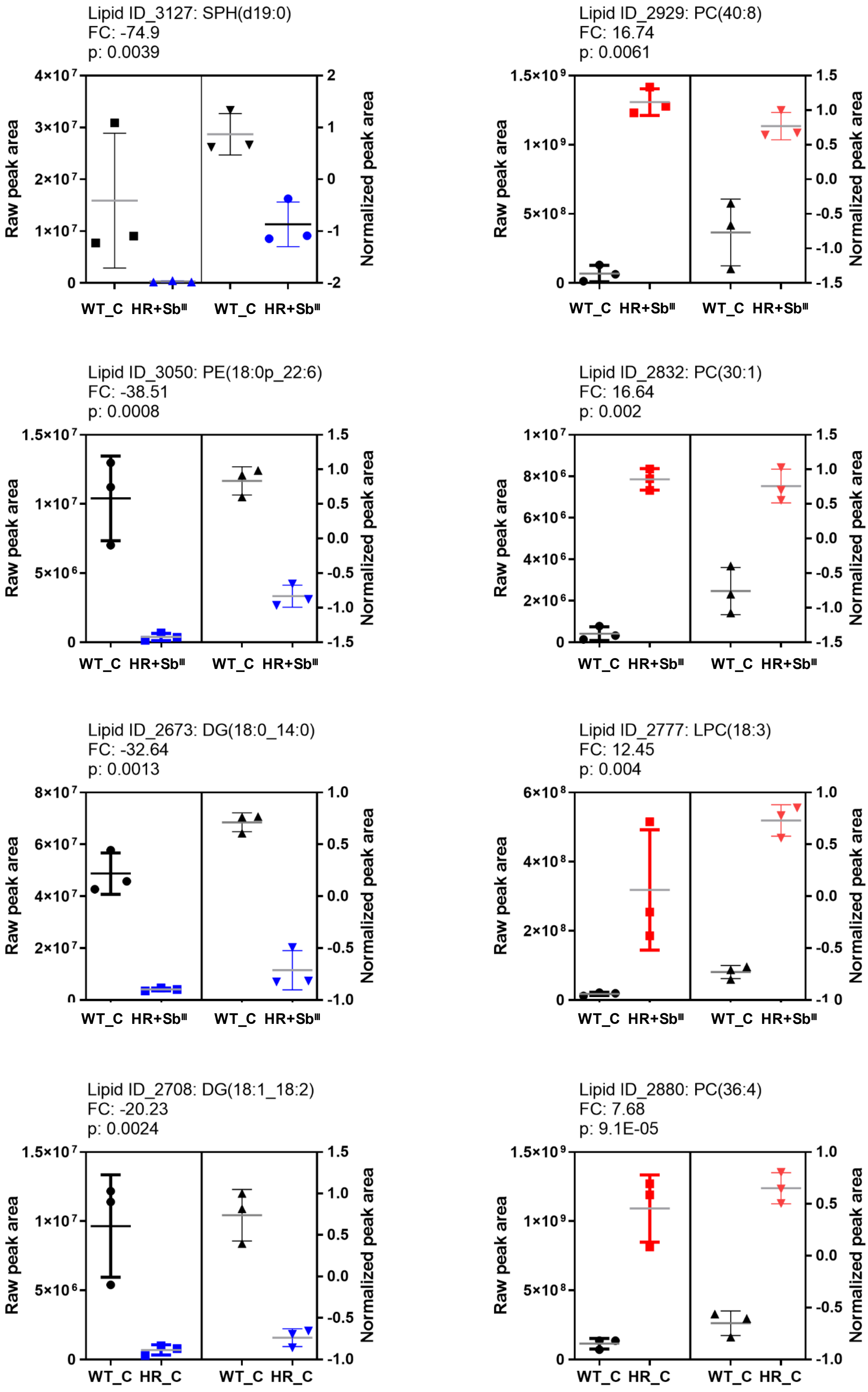

Supplementary figure 1. Potential biomarkers detected after OPLS-DA for discrimination between resistant and sensitive parasites without drug challege. The antimony resistant parasites (HR_C) were compared with sensitive parasites (WT_C) in absence of the drug. The downregulated and up-regulated potential lipid biomarkers are highlighted in blue or red, respectively. Raw and normalized peak area intensity are plotted by lipid compound. The fold change (FC) and the raw p-value (p) are included per lipid.

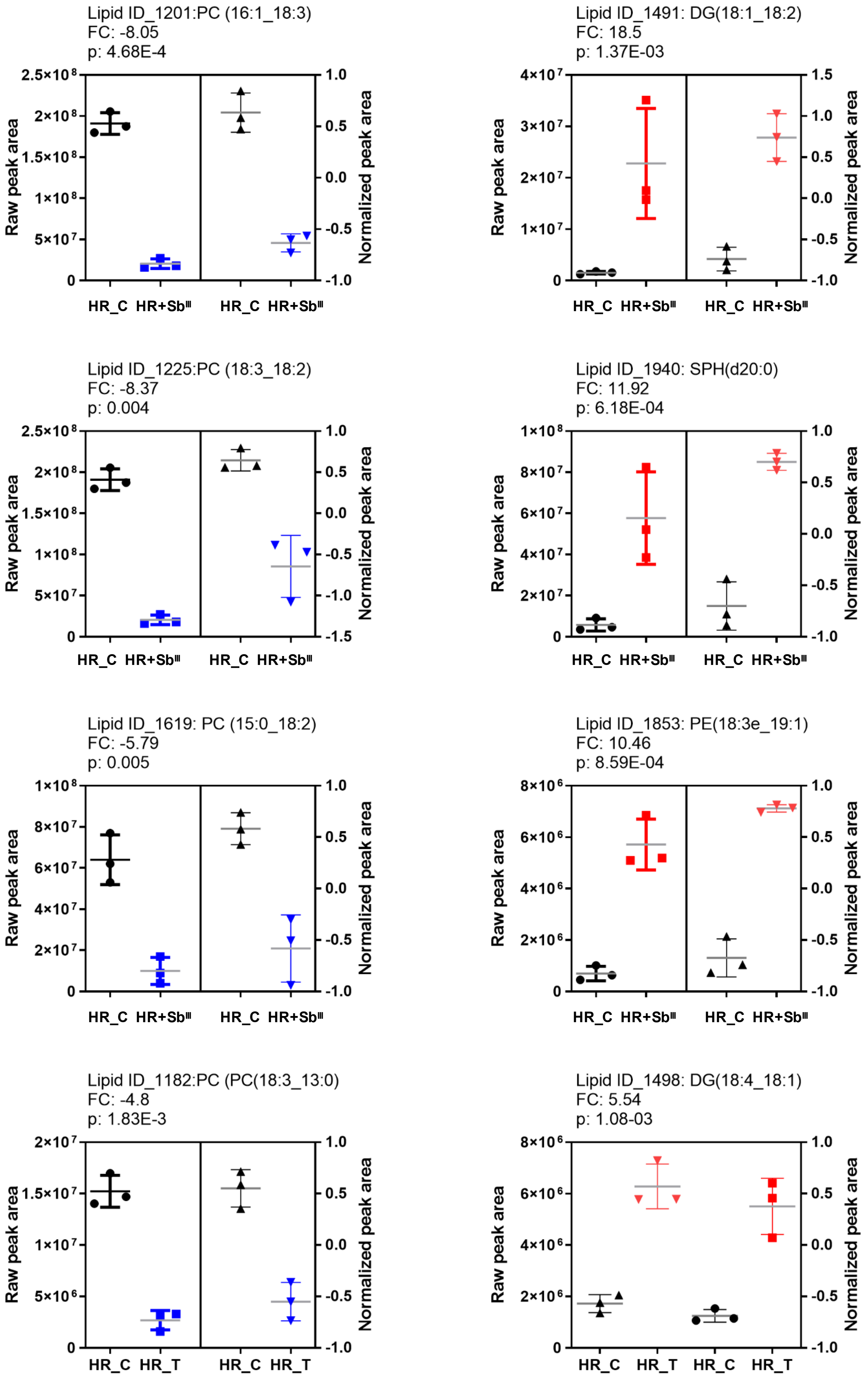

Supplementary figure 2. Potential biomarkers detected after OPLS-DA associated with the antimony resistant phenotype under drug challenge. The antimony resistant parasites growing under antimony challenge (HR+SbIII) were compared with the group of untreated parasites (HR_C). The downregulated and up-regulated potential lipid biomarkers are highlighted in blue or red, respectively. Raw and normalized peak area intensity are plotted by lipid compound. The fold change (FC) and the raw p-value (p) are included per lipid.

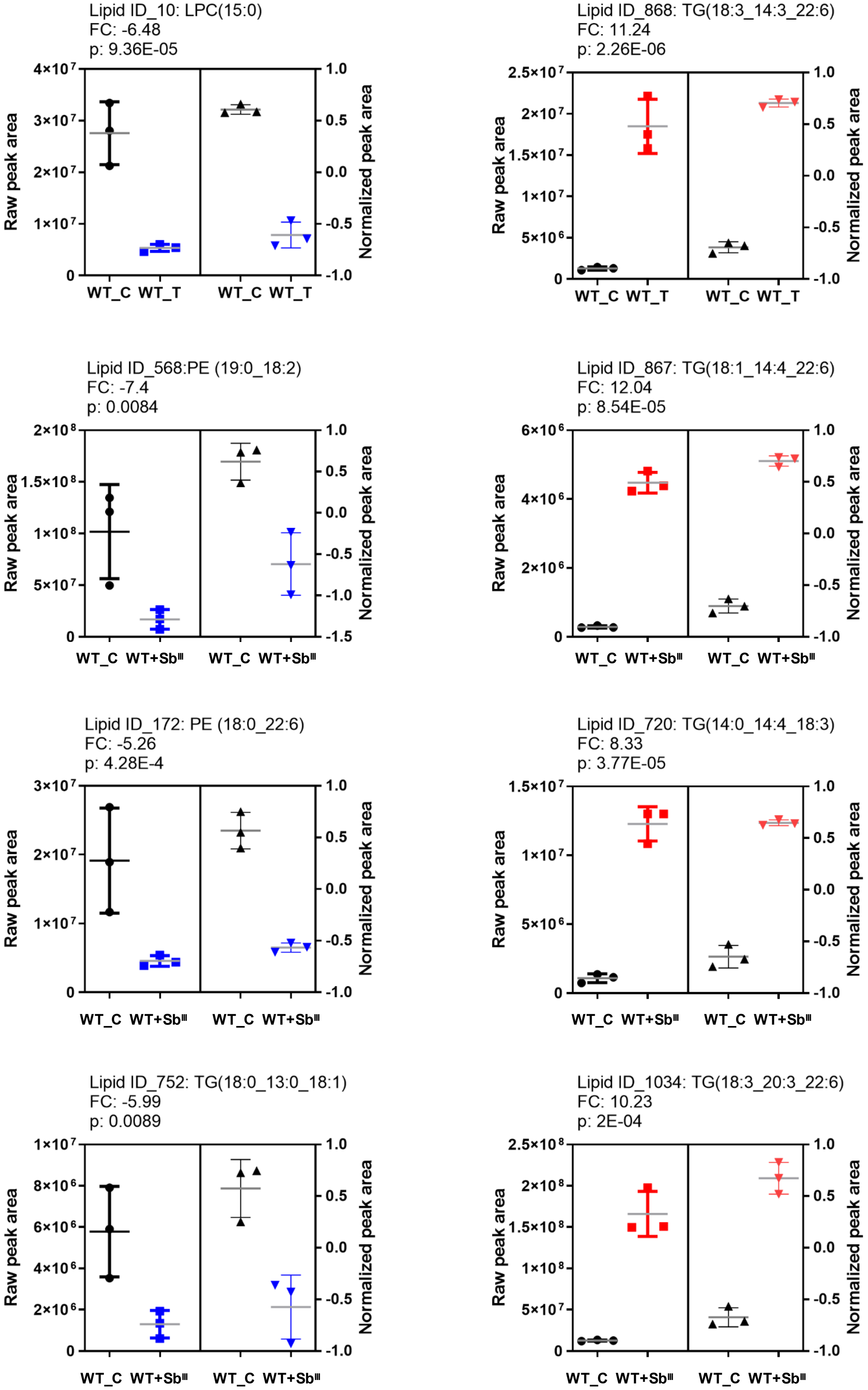

Supplementary figure 3. Potential biomarkers detected after OPLS-DA associated with the sensitive phenotype under drug pressure. The antimony sensitive parasites growing under antimony challenge (WT+SbIII) were compared with the group of untreated parasites (WT_C). The downregulated and up-regulated potential lipid biomarkers are highlighted in blue or red, respectively**.** Raw and normalized peak area intensity are plotted by lipid compound. The fold change (FC) and the raw p-value (p) are included per lipid.
